# Identifying Effective Components of Child Maltreatment Interventions: A Meta-analysis

**DOI:** 10.1007/s10567-017-0250-5

**Published:** 2017-12-04

**Authors:** Claudia E. van der Put, Mark Assink, Jeanne Gubbels, Noëlle F. Boekhout van Solinge

**Affiliations:** Research Institute of Child Development and Education, University of Amsterdam, P.O. Box 15780, 1001 NG Amsterdam, The Netherlands

**Keywords:** Child maltreatment, Child abuse, Intervention, Prevention, Effectiveness, Effective components, Meta-analysis

## Abstract

There is a lack of knowledge about specific components that make interventions effective in preventing or reducing child maltreatment. The aim of the present meta-analysis was to increase this knowledge by summarizing findings on effects of interventions for child maltreatment and by examining potential moderators of this effect, such as intervention components and study characteristics. Identifying effective components is essential for developing or improving child maltreatment interventions. A literature search yielded 121 independent studies (*N* = 39,044) examining the effects of interventions for preventing or reducing child maltreatment. From these studies, 352 effect sizes were extracted. The overall effect size was significant and small in magnitude for both preventive interventions (*d* = 0.26, *p* < .001) and curative interventions (*d* = 0.36, *p* < .001). Cognitive behavioral therapy, home visitation, parent training, family-based/multisystemic, substance abuse, and combined interventions were effective in preventing and/or reducing child maltreatment. For preventive interventions, larger effect sizes were found for short-term interventions (0–6 months), interventions focusing on increasing self-confidence of parents, and interventions delivered by professionals only. Further, effect sizes of preventive interventions increased as follow-up duration increased, which may indicate a sleeper effect of preventive interventions. For curative interventions, larger effect sizes were found for interventions focusing on improving parenting skills and interventions providing social and/or emotional support. Interventions can be effective in preventing or reducing child maltreatment. Theoretical and practical implications are discussed.

## Introduction

Child maltreatment is a serious problem that affects many children around the world. A recent series of meta-analyses showed that worldwide prevalence rates of child maltreatment ranged from 0.3% based on studies using maltreatment reports of professionals to 36.6% based on self-report studies (Stoltenborgh et al. 2014). Child maltreatment is associated with serious short-term and long-term negative consequences, such as physical, behavioral, and psychological problems, leading to high costs for individuals and society (Alink et al. 2012; Gilbert et al. 2008; Jonson-Reid et al. 2012) . Given the high prevalence rates and serious consequences of child maltreatment, effective prevention of child maltreatment is essential. The number of interventions aimed at preventing or reducing child maltreatment has grown over the years. However, several meta-analyses on the effectiveness of these interventions showed only a limited effect (e.g., Euser et al. 2015; Geeraerts et al. 2004; MacLeod and Nelson 2000; Sweet and Appelbaum 2004). Therefore, it is very important to unravel intervention components that contribute to intervention effectiveness from components not contributing to (or even negatively affecting) intervention effectiveness. Consequently, new promising interventions may be developed and existing interventions may be improved by integrating effective components in interventions and/or eliminating ineffective components from interventions. To enhance knowledge on how specific components of child maltreatment interventions affect the effectiveness of these interventions, a comprehensive meta-analysis was performed in the present study.

The number of interventions aimed at preventing or reducing child maltreatment has increased exponentially over the last decades (Daro 2011). Most interventions target a clearly defined population that is identified on the basis of risk factors for child maltreatment, such as teenage mothers, drug or alcohol addicted parents, or multiproblem families. Interventions may be aimed at *reducing* the incidence of child maltreatment in maltreating families or at *preventing* the occurrence of child maltreatment in at-risk, but non-maltreating families. The former are often system interventions, whereas the latter often comprise home visitation interventions in which parents are visited at home and provided with information, support, and/or training regarding child health, development, and care. These interventions often begin prenatally and continue during the child’s first 2 years of life, but may also begin after birth and last only a few months. In addition, some interventions are aimed at preventing the first occurrence of child maltreatment in the general population, for example by providing a short parental skills training to parents who visit a well-baby clinic.

A number of meta-analyses have synthesized results on the effectiveness of interventions aimed at preventing or reducing child maltreatment (e.g., Euser et al. 2015; Filene et al. 2013; Geeraerts et al. 2004; Guterman 1999; Layzer et al. 2001; Pinquart and Teubert 2010; Sweet and Appelbaum 2004). These studies generally found minor effects of interventions for reducing or preventing child maltreatment. In specific, Geeraerts et al. (2004) found a small effect (*d* = .29) of early prevention interventions for families with young children at risk for physical child abuse and neglect. Filene et al. (2013) found a small effect (*d* = .20) of home visitation interventions, and Pinquart and Teubert (2010) found a very small but significant effect (*d* = .13) of parent education interventions for expectant and new parents. Euser et al. (2015) also found a very small but significant effect (*d* = 0.13) of interventions aimed at preventing or reducing child maltreatment. However, this last effect was no longer significant after publication bias was taken into account by applying the trim-and-fill approach.

A few meta-analyses have attempted to identify characteristics of child maltreatment interventions associated with intervention effectiveness by examining potential moderators of the mean effect of interventions. However, none of these meta-analyses extensively examined whether and how specific intervention components, such as different content or delivery methods, influence intervention effectiveness. In studies on child maltreatment interventions, different terms are used for intervention components, such as practice elements (Chorpita and Daleiden 2009), kernels (Embry and Biglan 2008), behavior change techniques (Michie et al. 2013), and core components (Blase and Fixsen 2013). Blase and Fixsen (2013) classify core components into: (1) contextual factors, such as types of families served (e.g., low-income teenage parents, parents with alcohol or drug problems) and delivery settings (e.g., clinic-based therapy, home visits), (2) structural elements, such as the required number and specific sequence of sessions, and (3) specific intervention practices, such as teaching problem-solving behavior, practicing communication skills, and reinforcing appropriate behavior. Specific intervention practices can be further classified based on intervention content (such as increasing knowledge of typical child development, increasing parenting self-efficacy, and improving discipline and/or behavior management strategies) and delivery techniques used to engage parents and teach relevant content (such as group discussions, homework assignments, role-playing, and modeling).

The main objective of this meta-analysis was to determine whether and how intervention components (i.e., intervention content and delivery techniques) influence the effectiveness of child maltreatment interventions. In doing so, important insight can be gained into what components contribute to intervention effectiveness and, consequently, how child maltreatment interventions can best be designed to reach optimal effectiveness. For this purpose, we conducted a three-level meta-analysis in which we tested intervention components as potential moderators of the (mean) effect of child maltreatment interventions. As we aimed for a comprehensive meta-analysis, we included (a) two types of interventions: *preventive* interventions targeting the general population or families at risk for child maltreatment and *curative* interventions targeting maltreating families that are aimed at reducing maltreatment, (b) randomized controlled trials (RCTs) as well as high quality quasi-experimental studies, and (c) recently conducted studies, as previous meta-analyses included studies that were published until 2013.

Determining which components appear to be essential (or nonessential) across a variety of interventions aimed at reducing or preventing child maltreatment has important implications for clinical practice. First, when choosing among interventions to implement, such information could be used to select interventions containing components associated with greater intervention effectiveness. Second, the effectiveness of existing interventions could be improved by integrating specific components associated with greater effectiveness into interventions. Third, it may be possible to eliminate components associated with less effective interventions, thereby minimizing the burden on practitioners and families.

In the present study, we aimed to examine the effect of contextual factors (i.e., general aim of the intervention, types of families served, delivery setting, type of intervention, specific intervention, age of the children), structural elements (i.e., type of worker, duration of intervention, number and interval of intervention sessions), content (parenting skills, personal skills parents, self-confidence of parents, attitudes/expectations toward parenting, knowledge of typical child development, social network of family, relationship between parents, relationship between parent and child, parental mental health problems, parental empowerment, social/emotional support, well-being child, child skills, practical support, motivation to change), and delivery methods (i.e., modeling, role-playing, monitoring, (psycho) education, homework assignments, cognitive skills training, and family group conferencing). We also examined the effect of study design characteristics such as sample characteristics (i.e., sample size, age of parents, percentage of cultural minorities), design of the study (randomized controlled trial versus quasi-experimental), and outcome characteristics (follow-up duration, type of outcome measure).

## Method

### Inclusion Criteria

Studies were selected if they met the following three criteria. First, studies had to report on the effect of at least one intervention for preventing or reducing child maltreatment. In specific, we included two types of interventions: *preventive* interventions targeting the general population or targeting families at risk for child maltreatment and *curative* interventions targeting maltreating families aimed at reducing maltreatment or recurrence of maltreatment. In this review, we followed the definition of child maltreatment as formulated by the Centers for Disease Control and Prevention: “any act or series of acts of commission or omission by a parent or other caregiver that results in harm, potential for harm, or threat of harm to a child.” Given this broad definition, we included studies that reported on interventions for physical abuse, sexual abuse, and neglect. In addition, studies examining the effect of child maltreatment interventions on harsh parenting (such as corporal/physical punishment or parental aggression toward children) and out-of-home placement were also included. Second, experimental studies as well as quasi-experimental studies (in which a treatment condition is compared to a control condition) were included. Although randomized controlled trials can be regarded as the “golden standard” for studies examining the effectiveness of interventions (Farrington 2003), we decided to also include quasi-experimental studies, since RCTs are rather scarce in the field of child protection due to practical and ethical concerns for true experimental designs. Third, studies had to report at least one effect size or sufficient information to calculate at least one effect size.

### Selection of Studies

The electronic databases PsychINFO, ERIC, PubMed, Web of Science, ScienceDirect, and Google Scholar were searched for articles, books, chapters, dissertations, and reports. Until March 2017, studies were collected using keywords regarding study design, intervention features, study outcomes, and participants in different combinations: ‘(quasi-)experiment’, ‘randomized control*’, ‘trial’, ‘RCT’ ‘child*’, ‘abus*’, ‘maltreat*’, ‘neglect*’, ‘interven*’, ‘preven*’, ‘home visit*’, ‘recur*’, ‘recidiv*’, ‘relaps*’, ‘family group conferencing’ ‘randomized’, ‘evaluat*’, and ‘experiment*’.

**Fig. 1:**
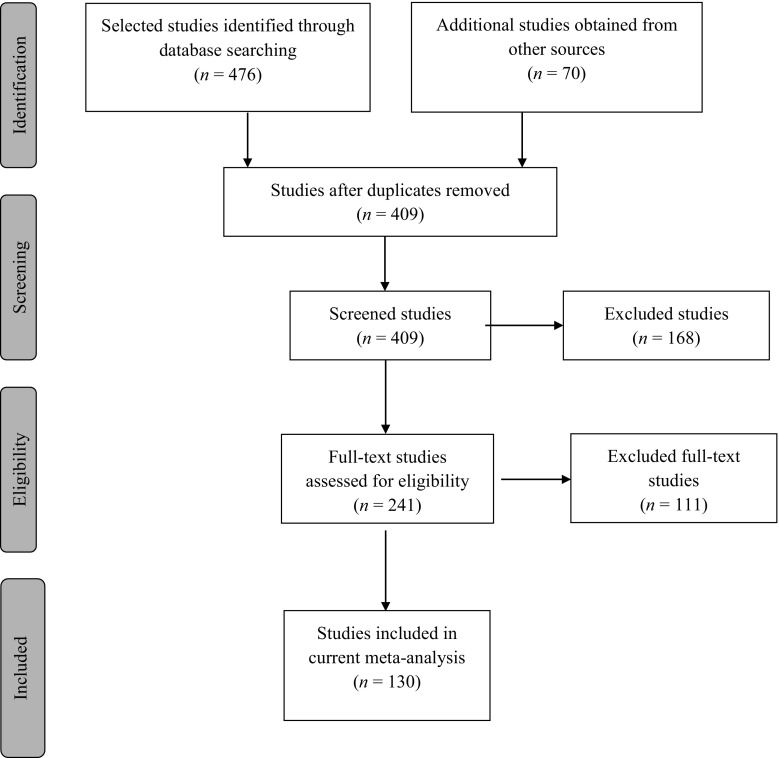
Flowchart of selection of studies for inclusion in meta-analysis

Next, manual searches of reference sections of the retrieved articles, reviews, and book chapters were conducted. Finally, we contacted authors by email to request for studies and unpublished manuscripts on the effect of child maltreatment interventions that may be relevant for inclusion in the present review. The search procedure is depicted in the flow diagram presented in Fig. 1. The search yielded 546 relevant studies of which 130 studies met the inclusion criteria.

### Study Coding

Following the guidelines of Lipsey and Wilson (2001), a coding scheme was developed to code all study design and intervention characteristics that could moderate the effect of child maltreatment interventions.

First, with regard to *study design characteristics*, we collected information on publication year, sample size, parental age, percentage of cultural minorities in samples, research design (RCT, quasi-experimental design), pilot/feasibility study (yes/no), follow-up length (in months), and type of outcome measure (self-report of parents, official reports, observations, hospital data, self-report of children). Second, we retrieved information on intervention characteristics, which we divided into contextual factors, structural elements, and specific intervention practices (content and delivery techniques). The *contextual factors* included the general aim of the intervention (reducing risk for child maltreatment versus reducing actual child maltreatment), type of families served (maltreating parents, general population, risk group identified by means of a questionnaire, risk group identified based on the presence of one or two risk factors), delivery setting (home/ambulant, treatment center, combination of locations), post-/prenatal intervention (postnatal, prenatal, postnatal and prenatal), type of intervention (cognitive behavioral therapy, home visitation interventions [in which parents are visited at home and provided with information, support and/or training regarding child health, development and care], parent training interventions [aimed at learning specific parenting skills], family-based/multisystemic interventions [aimed at the whole family system/multiple social systems], substance abuse intervention [primarily targeting substance abuse of parents], before-school interventions, general prevention interventions [aimed at preventing the occurrence of child maltreatment in the general population], crisis interventions [aimed at solving acute problems], and combined interventions), specific individual interventions (ACT Parenting Raising Safe Kids, (Early) Head Start, PCIT, FGC/FCDM, Healthy Families, Healthy Start, Healthy Steps, Intensive Family Preservation Services, Incredible Years, MST (CAN/BSF), Nurse Family Partnership (NFP), Triple P, Parents as Teachers, Project 12-Ways, Safe Environment for Every Kid, Child Parent Enrichment Program, SafeCare, other), and age of the child (unborn child/baby (< 2), infant/toddler (2–5), primary school (6–12), high school (> 12). The *structural elements* included type of worker (professionals only versus professionals and others), the duration of the intervention (0–6 months, 7–12 months, 13–24 months, > 24 months), the minimum duration of the intervention, the maximum duration of the intervention, the average number of sessions, and the interval of the sessions (weekly, multiple sessions a week, every other week/monthly, ascending/descending intensity, every 3 months). The *specific intervention practices (content)* included parenting skills (yes/no), personal skills of parents (yes/no), parents’ self-confidence (yes/no), attitudes or expectations about parenting (yes/no), knowledge of typical child development (yes/no), social network of the family (yes/no), relationship between parents (yes/no), relationship between parent and child (yes/no), mental health problems parents (yes/no), parental empowerment (yes/no), social or emotional support (yes/no), well-being of the child (yes/no), child skills (yes/no), practical support (yes/no), and motivation (yes/no). Finally, we examined the following delivery techniques: modeling (yes/no), role-playing (yes/no), monitoring (yes/no), (psycho)education (yes/no), homework assignments (yes/no), cognitive skills training (yes/no), and family group conferencing (yes/no). Inter-rater agreement was based on a double-coding of 14 studies by two independent coders. An inter-rater agreement of 97% was found between the two coders on all coded variables.

### Calculation of Effect Sizes

All outcomes of the primary studies were transformed into the standardized difference between two means, also referred to as Cohen’s *d.* The effect sizes were calculated using formulas of Ferguson (1966), Lipsey and Wilson (2001), and Rosenthal (1994). In most instances, proportions, means and standard deviations, and odd ratios were transformed into Cohen’s *d*. If insufficient statistical information was provided for calculating an effect size, we contacted the study authors and asked for the required information. In calculating each effect size, it was important that the direction of the effect (positive or negative) corresponded with the statistical data reported in the primary study. A positive effect indicated less child maltreatment (or levels of other factors, such as harsh parenting) was found in the intervention group than in the control group, whereas a negative effect indicated more child maltreatment in the intervention group than in the control group. If results were reported to be nonsignificant and additional statistical information required for calculating an effect size was not reported, the value of zero was assigned to an effect and added to the data set (Durlak and Lipsey 1991).

All coded data and calculated effect sizes were entered in SPSS version 22. Before the analyses were performed, continuous variables were centered around their mean, and categorical variables were recoded into dummy variables for each category of a variable. Further, extreme values of effect sizes and sample sizes (*Z* > 3.29 or *Z* < − 3.29; Tabachnik and Fidell 2013) were identified.

### Statistical Analyses

For estimating the overall effect for all included child maltreatment interventions, only preventive interventions, and only curative interventions, as well as for examining potential moderating variables, a three-level meta-analysis technique was used. By applying a multilevel approach to meta-analysis, there is no need for aggregating or selecting data, implying that all relevant effect sizes can be extracted from primary studies (see also Assink et al. 2015; Assink and Wibbelink 2016). As a result, all information from primary studies is preserved and maximum statistical power can be achieved. In our meta-analytic model, three forms of variance were taken into account: random sampling variation of observed effect sizes (level 1), variance within studies (level 2), and variance between studies (level 3) (Cheung 2014; Hox 2002; Van den Noortgate et al. 2013, 2014). The sampling variance (level 1) was not estimated, but considered to be known and calculated using the formula of Cheung (2014, p. 2015). Because we considered the primary studies to be a random sample from a larger population of studies, we built random-effects models.

Estimating the overall effects was done in separate three-level intercept-only models. Effect sizes were weighted by the inverse of their variance (i.e., sampling error), so that effect sizes derived from studies using samples of larger size contributed more to the overall effect size estimate than effect sizes derived from studies using samples of smaller size. Next, the significance of the variance distributed on levels 2 and 3 were tested by conducting two separate one-tailed log-likelihood ratio tests. In these tests, the deviance of a model in which the variance on either level 2 or level 3 was set to zero, was compared to the deviance of the full model in which level 2 and level 3 variances were freely estimated. In case the level 2 and/or level 3 variance was significant, the distribution of effect sizes was considered to be heterogeneous. This indicates that the effect sizes could not be treated as estimates of one common effect size, and thus, moderator analyses were performed to search for variables that can explain the variance. Potential moderating variables (i.e., study design characteristics, contextual factors, structural elements, and specific intervention practices) were examined by testing them in a three-level meta-analytic model as covariates. In all meta-analytic models, the Knapp and Hartung correction (Knapp and Hartung 2003) was applied, implying that the significance of coefficients was tested using the *t*- and *F*- distributions rather than the *z*-distribution.

The statistical software package R (version 3.2.0) and the metafor package (Viechtbauer 2010) were used to build the 3-level meta-analytic models. We used the syntax as described by Assink and Wibbelink (2016). In all analyses, a 5% significant level was used.

### Publication Bias and Sensitivity Analyses

A common problem in conducting a meta-analysis is that studies with nonsignificant or negative results are less likely to be published than studies with positive and significant results. This phenomenon is called publication bias and is often referred to as the ‘file drawer problem’ (Rosenthal 1995). So, the effects extracted from primary studies included in this meta-analysis may not be an adequate representation of the actual effect of child maltreatment interventions. Besides publication bias, the results may be affected by other forms of bias, such as coding or selection bias. Therefore, we examined the degree to which our results were affected by (different forms of) bias by conducting the nonparametric and funnel plot-based trim-and-fill analysis as described by Duval and Tweedie (2000a, b). A funnel plot is a scatter plot of the effect sizes against the effect size’s precision (1 divided by the standard error). In this analysis, the symmetry of the funnel is tested, as the plot would be asymmetric when bias is present. In case of an asymmetric plot, the asymmetry is restored by imputing “missing” effect sizes that are estimated on the basis of existing effect sizes in the data set. Subsequently, a “corrected” overall effect can be estimated in a sensitivity analysis using the data set to which the imputed effect sizes produced by the trim-and-fill algorithm have been added. In this way, the degree to which the results were affected by bias can be made visible.

As we identified seven outlying effect sizes (see above), we performed a sensitivity analysis in which we estimated an overall effect of child maltreatment interventions using the data in which the outlying effect sizes were excluded. In this way, we could determine the degree to which the initially estimated overall effect was robust against outliers in effect sizes. In addition, we estimated the overall effect of child maltreatment interventions excluding the results of pilot/feasibility studies, because these studies are more likely to show an effect that is larger than effects produced by well-powered trials.

## Results

### Descriptive Characteristics

The present meta-analysis included 130 studies with *k* = 121 non-overlapping samples, comprising *N* = 39.044 participants. A total of 352 effect sizes were extracted from all primary studies, which examined the effect of home visitation interventions (*k* = 50), parent training interventions (*k* = 29), family-based/multisystemic interventions (*k* = 17), substance abuse interventions (*k* = 4), before-school interventions (*k* = 4), general prevention interventions (*k* = 4), crisis interventions (*k* = 3), cognitive behavioral therapy (*k* = 2), and combined interventions (*k* = 15). These studies were published between 1977 and 2017 (with 2007 being the median publication year) and were conducted in the USA (*k* = 93), Europe (*k* = 11), Canada (*k* = 6), Australia or New Zealand (*k* = 6), and in various other countries (*k* = 5). The characteristics of the studies are presented in “Appendix 1,” including the name of the child maltreatment intervention, publication year, sample size, age of the child at the start of the intervention, study design (RCT or quasi-experimental), and type of families served (risk group, general population, or maltreating parents). The distribution of the moderators for preventive and curative interventions is presented in Table 1.

**Table 1:** Distribution of moderators separately for preventive and curative interventions

	Preventive interventions (*n* = 91) (%)	Curative interventions *(n* = 32) (%)	*χ* ^2^(*df*)
Research design			1.31(1)
Randomized controlled trial	68.1	56.7	
Quasi-experimental	31.9	43.4	
Type of outcome measure			3.21(2)
Self-report parents	58.2	43.3	
Official reports	35.2	53.3	
Other (observations, self-report child)	6.6	3.3	
Type of worker			14.90(2)***
Only professionals	48.8	89.7	
Professionals and others	51.2	10.3	
Delivery setting			1.03(2)
Home/ambulant	54.7	48.0	
Treatment center	22.1	32.0	
Combination of locations	23.3	20.0	
Age of the child			42.5(2)***
2 years or younger	67.8	7.7	
2–5 years old	21.8	23.1	
6 year or older	10.3	69.2	
Duration			13.5(3)*
0–6 months	38.8	58.3	
7–12 months	5.9	20.8	
13–24 months	25.9	20.8	
> 24 months	29.4	0.0	
Type of intervention			51.1(7)***
Home visitation intervention	48.4	16.7	
Parenting training	25.3	20.0	
Family-based/multisystemic	4.4	46.7	
Substance-abuse intervention	0.0	10.0	
Before-school intervention	3.3	0.0	
General prevention intervention	4.4	0.0	
Crisis intervention	1.1	6.7	
Combined interventions	13.2	0.0	
Specific intervention practices			
Improving parental skills	76.9	70.0	.58(2)
Improving personal skills parents	38.5	70.0	9.03(2)**
Increasing parent’s self-confidence	19.8	20.0	.01(2)
Improving attitudes/expectations toward parenting	11.0	3.3	1.60(2)
Improving knowledge of typical child development	63.7	13.3	22.94***
Strengthening social network	27.5	43.3	2.64(2)
Improving relationship between parents	13.2	20.0	.83(2)
Improving relationship between parent– child	50.5	46.7	.14(2)
Addressing parental mental health problems	23.1	36.7	2.14(2)
Empowerment	23.1	33.3	1.25(2)
Social/emotional support	49.5	23.3	6.28(2)*
Improving well-being child	51.6	40.0	1.23(2)
Improving child skills	15.4	20.0	.35(2)
Practical support	27.5	33.3	.38(2)
Improving motivation	3.3	23.3	11.95(2)**
Delivery techniques			
Modeling	34.6	22.2	1.43(2)
Discussion	44.9	40.7	.14(2)
Role-playing	11.5	33.3	6.71(2)**
Monitoring	23.1	18.5	.24(2)
Psycho(education)	71.8	25.9	17.58(2)***
Homework assignments	9.0	22.2	3.25(2)^+^
Cognitive skills training	3.8	25.9	11.35(2)**
Family group conferencing	2.6	22.2	11.01(2)***

*df* Degrees of freedom

* *p* < .05; **p < .01; *** *p* < .001

^+^ *p* < .1

## Overall Effect, Heterogeneity in Effect Sizes, and Sensitivity Analyses

A significant overall effect was found of interventions aimed at preventing or reducing child maltreatment with a Cohen’s *d* of 0.287; 95% CI [0.226; 0.348], *t*(351) = 9.209, *p* < .001 (see Table 2). According to the criteria formulated by Cohen (1988), this effect is small in magnitude. The two log-likelihood ratio tests showed that significant variance was present both at level 2 (*χ* ^2^(1) = 178.015, *p* < .001; one-sided) and level 3 (*χ* ^2^(1) = 48.182, *p* < .001; one-sided) of the meta-analytic model. Of the total variance, 32.8 and 56.1% were distributed at levels 2 and 3, respectively, and 11.1% was the percentage of sampling variance that was calculated using the formula of Cheung (2014, p. 2015).

**Table 2:** Overall Effect for all interventions, preventive interventions, curative interventions, and all interventions excluding pilot/feasibility studies

	# Studies	# ES	Mean *d* (SE)	95% CI	Sig. mean *d* (*p*)	% Var. at level 1	Level 2 variance	% Var. at level 2	Level 3 variance	% Var. at level 3
All interventions	121	352	0.287 (0.031)***	0.226, 0.348	< .001***	11.11	0.043***	32.76	0.073***	56.13
Preventive interventions	91	217	0.263 (0.034)***	0.197, 0.329	< .001***	9.44	0.028***	26.43	0.067***	64.13
Curative interventions	32	135	0.364 (0.069)***	0.227, 0.502	< .001***	21.26	0.147***	55.70	0.061*	23.04
All interventions excluding pilot/feasibility studies	114	332	0.278 (0.032)***	0.215, 0.341	< .001***	10.56	.045***	34.22	.072***	55.22

# *Studies* number of studies; # *ES* number of effect sizes, *Mean d* mean effect size (Cohen’s *d*), *SE* standard error, *CI* confidence interval, *Sig* significance,  *% Var* percentage of distributed variance, *level 1* variance sampling variance, *level 2 variance* variance within studies, *level 3 variance* variance between studies

*p < .05; ***p < .001

**Fig. 2:**
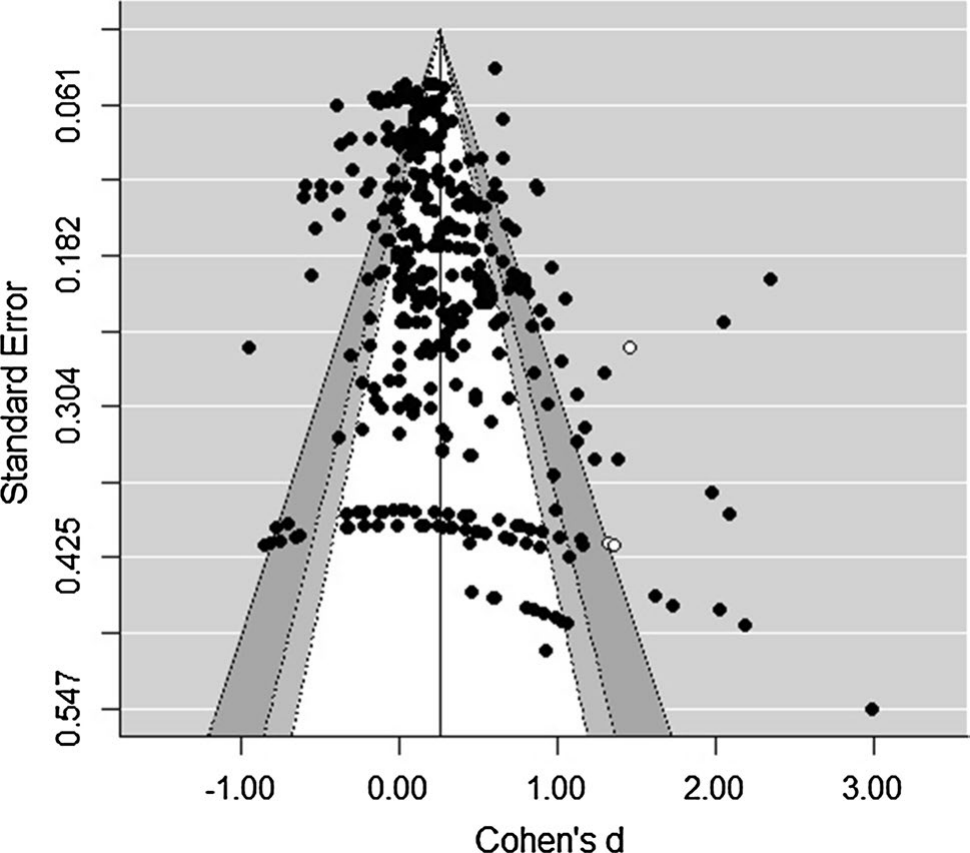
Funnelplot

The results of the trim-and-fill analysis showed that bias may be present in the data, as the distribution of all effect sizes (produced by both preventive and curative interventions) was asymmetrical. Figure 2 shows that a small number of effect sizes had to be imputed in the right side of the funnel to restore its symmetry. In specific, 3 effect sizes from 2 independent studies were added to the data and after re-estimating the overall effect, a slightly higher (Δ*d* = 0.014) significant effect was found (*d* = 0.301 [95% CI 0.238; 0.365], *t*(354) = 9.343, *p* < .001). Besides asymmetry in the distribution of effect sizes, there were a number of effect sizes that seemed to be “outliers” compared with other effect sizes (i.e., the effects in the most right part of Fig. 2). To determine whether and how these “outlying” effect sizes influenced our estimated overall effect, we performed a sensitivity analysis in which we re-estimated an overall effect after excluding 7 effect sizes with a *Z* score exceeding 3.29 (Tabachnik and Fidell 2013) from the data. The results produced an overall effect of *d* = 0.257; 95% CI [0.204, 0.311], *t*(344) = 9.499, *p* < .001, which is slightly below our initial estimated overall effect (Δ*d* = 0.030). In addition, we estimated the overall effect of all interventions using data without effects derived from the pilot studies (see Table 2). The influence of pilot/feasibility studies on the overall summary effect was only small (*d* = 0.287 with pilot/feasibility studies and *d* = 0.278 without pilot/feasibility studies; Δ*d* = 0.009). All three sensitivity analyses showed that the estimated overall effect of *d* = 0.287 was rather robust.

Next, we estimated an overall effect of only preventive interventions, and this yielded a Cohen’s *d* of 0.263; 95% CI [0.197; 0.329], which is a small effect according to the criteria of Cohen (1988). The two log-likelihood ratio tests showed once again that significant variance was present at level 2 (*χ* ^2^(1) = 141.121, *p* < .001; one-sided) and level 3 (*χ* ^2^(1) = 45.038, *p* < .001; one-sided) of the meta-analytic model. Of the total variance, 26.4 and 64.1% were distributed at levels 2 and 3, respectively, and 9.4% was distributed at level 1. As these results indicated substantial heterogeneity in effect sizes, we could test study design and intervention characteristics as potential moderators of the effect of preventive interventions. The estimated overall effect of curative interventions was also significant with a Cohen’s *d* of 0.364; 95% CI [0.227; 0.502] and was small in magnitude according to Cohen’s criteria. Both the level 2 variance (*χ* ^2^(1) = 55.467, *p* < .001; one-sided) and the level 3 variance (*χ* ^2^(1) = 3.975, *p* = .023; one-sided) were significant, and of the total variance, 21.3, 55.7, and 23.0% were distributed at levels 1, 2, and 3, respectively. So, also for curative interventions, moderator analyses could be performed to examine whether and how the intervention effect was influenced by study design and intervention characteristics.

### Moderator Analyses Bivariate Models

Table 3 shows the results of the moderator analyses performed for the preventive interventions, and Table 4 shows the results of the moderator analyses performed for the curative interventions.

**Table 3:** Results of the moderator analyses for preventive interventions (bivariate models)

Moderator variables	# Studies	# ES	Intercept/mean *d* (95% CI)	*β*_1_ (95% CI)	*F* (*df*1, *df*2)^a^	*p* ^b^	Level 2 variance	Level 3 variance
Overall effect	91	217	0.263 (0.197, 0.329)***					
**A: Study design characteristics**								
Publication year	91	217	0.264 (0.197, 0.330)***	0.002 (− 0.005, 0.009)	0.211 (1, 215)	.646	.028***	.068***
*Sample characteristics*								
Sample size	91	217	0.263 (0.197, 0.330)***	− 0.000 (− 0.000, 0.000)	2.559 (1, 215)	.111	.027***	.069***
Age of the parents	18	49	0.377 (0.239, 0.515)***	0.013 (− 0.015, 0.040)	0.865 (1, 47)	.357	.000	.060***
Percentage cultural minorities	63	163	0.817 (− 0.084, 1.718)^+^	0.190 (− 0.084, 0.464)	1.867 (1, 161)	.174	.023***	.063***
*Design characteristics*								
Research design					3.720 (1, 215)	.055^+^	.028***	.064***
RCT (RC)	63	159	0.226 (0.151, 0.301)***					
Quasi-experimental	29	58	0.347 (0.238, 0.455)***	0.121 (− 0.003, 0.245)^+^				
Pilot/feasibility study					0.009 (1, 215)	.922	.028***	.068***
No (RC)	87	213	0.263 (0.195, 0.330)***					
Yes	4	4	0.282 (− 0.111, 0.676)	0.020 (− 0.380, 0.419)				
*Outcome characteristics*								
Follow-up period	40	80	0.261 (0.150, 0.372)***	0.002 (0.000, 0.003)*	5.480 (1, 78)	.022*	.014***	.092***
Type of outcome measure					1.727 (2, 214)	.180	.027***	.068***
Self-report parents (RC)	62	147	0.296 (0.221, 0.371)***					
Official reports	41	58	0.213 (0.120, 0.305)***	− 0.083 (− 0.179, 0.012)^+^				
Other	7	12	0.200 (− 0.012, 0.413)^+^	− 0.095 (− 0.311, 0.120)				
**B: Contextual factors**								
Type of families served					0.686 (2, 214)	.505	.028***	.069***
Risk group (questionnaire) (RC)	36	104	0.308 (0.205, 0.411)***					
Risk group (risk factor)	49	95	0.226 (0.133, 0.319)***	− 0.082 (− 0.221, 0.057)				
General population	8	18	0.266 (0.061, 0.472)***	− 0.042 (− 0.271, 0.188)				
General aim of the intervention					0.014 (1, 215)	.906	.028***	.069***
Reducing risk (RC)	85	199	0.262 (0.194, 0.331)***					
General prevention	8	18	0.275 (0.071, 0.479)**	0.013 (− 0.197, 0.223)				
Delivery setting					0.772 (2, 208)	.463	.026***	.072***
Home/ambulant (RC)	47	125	0.288 (0.195, 0.381)***					
Treatment center	19	45	0.273 (0.128, 0.418)***	− 0.015 (− 0.188, 0.158)				
Combination of locations	21	41	0.185 (0.046, 0.323)**	− 0.103 (− 0.270, 0.064)				
Post/prenatal					1.571 (2, 212)	.210	.028***	.065***
Postnatal (RC)	54	135	0.304 (0.219, 0.389)***					
Prenatal	4	11	0.293 (0.060, 0.527)*	− 0.010 (− 0.256, 0.235)				
Prenatal and postnatal	34	69	0.187 (0.080, 0.293)***	− 0.117 (− 0.253, 0.019)^+^				
Type of intervention					3.616 (7, 209)	.001***	.028***	.050***
Home visitation intervention (RC)	45	105	0.210 (0.126, 0.293)***					
Parent training	23	56	0.428 (0.305, 0.551)***	0.218 (0.070, 0.367)**				
Family-based/multisystemic	4	7	0.343 (0.027, 0.660)*	0.134 (− 0.194, 0.461)				
Substance-abuse intervention	1	1	1.852 (0.933, 2.770)***	1.642 (0.720, 2.564)***				
Before-school intervention	4	8	0.148 (− 0.117, 0.413)	− 0.062 (− 0.335, 0.212)				
General prevention	4	16	0.024 (− 0.219, 0.267)	− 0.186 (− 0.442, 0.071)				
Crisis intervention	1	2	0.407 (− 0.114, 0.929)	0.198 (− 0.330, 0.726)				
Combined intervention	15	22	0.174 (0.024, 0.324)*	− 0.036 (− 0.203, 0.131)				
Specific individual interventions					0.552 (13, 203)	.889	.027***	.080***
Other interventions (RC)	49	84	0.311 (0.211, 0.411)***					
ACT parents raising safe kids	3	6	0.377 (− 0.013, 0.768)^+^	0.066 (− 0.337, 0.469)				
(Early) head start	5	12	0.074 (− 0.208, 0.357)	− 0.237 (− 0.537, 0.063)				
Healthy families	10	35	0.189 (− 0.004, 0.382)^+^	− 0.123 (− 0.340, 0.095)				
Healthy start	4	12	0.339 (0.012, 0.666)*	0.028 (− 0.314, 0.369)				
Healthy steps	2	10	0.189 (− 0.235, 0.614)	− 0.122 (− 0.558, 0.314)				
Intensive fam preservation serv	1	2	0.407 (− 0.217, 1.030)	0.095 (− 0.536, 0.727)				
Incredible years	3	11	0.307 (− 0.085, 0.699)	− 0.005 (− 0.409, 0.400)				
NFP	2	7	0.298 (− 0.146, 0.741)	− 0.013 (− 0.468, 0.441)				
Triple P	4	18	0.417 (0.092, 0.741)*	0.105 (− 0.234, 0.445)				
Parents as teachers	2	4	0.079 (− 0.376, 0.535)	− 0.232 (− 0.698, 0.234)				
Safe environment for every Kid	2	7	0.058 (− 0.360, 0.475)	− 0.254 (− 0.683, 0.176)				
Child–parent enrichment progr.	2	4	0.102 (− 0.375, 0.580)	− 0.209 (− 0.697, 0.279)				
SafeCare	1	3	0.139 (− 0.491, 0.768)	− 0.173 (− 0.811, 0.465)				
Age of the child					1.162 (3, 200)	.325	.028***	.069***
Unborn child/baby (≤ 2) (RC)	59	132	0.222 (0.140, 0.304)***					
Infant/toddler (2–5)	19	53	0.365 (0.219, 0.512)***	0.143 (− 0.025, 0.311)^+^				
Primary school (6–12)	9	17	0.305 (0.104, 0.505)**	0.083 (− 0.127, 0.292)				
High school (≥ 12)	1	2	0.465 (− 0.188, 1.118)	0.243 (− 0.415, 0.902)				
**C: Structural elements**								
Type of worker					4.789 (1, 207)	.030*	.028***	.065***
Only professionals (RC)	41	109	0.334 (0.236, 0.432)***					
Professionals and others	46	100	0.184 (0.091, 0.277)***	− 0.150 (− 0.285, − 0.015)*				
Duration					4.484 (3, 199)	.005**	.021***	.047***
0–6 months (RC)	25	82	0.361 (0.268, 0.454)***					
7–12 months	5	8	− 0.051 (− 0.295, 0.192)	− 0.412 (− 0.673, − 0.152)**				
13–24 months	23	41	0.236 (0.119, 0.354)***	− 0.124 (− 0.273, 0.024)^+^				
> 24 months	25	72	0.190 (0.090, 0.289)***	− 0.171 (− 0.302, − 0.041)*				
Minimum duration (in weeks)	23	49	0.343 (0.198, 0.488)***	− 0.002 (− 0.004, 0.001)	2.303 (1, 47)	.136	.026***	.067*
Maximum duration (in weeks)	62	152	0.242 (0.173, 0.311)***	− 0.001 (− 0.002, − 0.000)*	6.337 (1, 150)	.013*	.020***	.043***
Average number of sessions	43	96	0.243 (0.145, 0.340)***	− 0.001 (− 0.008, 0.006)	0.133 (1, 94)	.716	.005	.077***
Interval sessions					1.345 (4, 153)	.256	.018***	.056***
Weekly (RC)	30	63	0.352 (0.244, 0.461)***					
Multiple sessions a week	4	8	0.325 (0.025, 0.626)*	− 0.027 (− 0.346, 0.292)				
Every other week/monthly	12	20	0.277 (0.119, 0.435)***	− 0.075 (− 0.267, 0.116)				
Ascending/descending intensity	25	62	0.185 (0.075, 0.295)**	− 0.167 (− 0.319, − 0.015)*				
Every 3 months	1	5	0.107 (− 0.380, 0.595)	− 0.245 (− 0.745, 0.254)				
**C: Specific intervention practices (content)**								
Parenting skills					1.513 (1, 215)	.220	.028***	.067***
No (RC)	21	48	0.188 (0.051, 0.325)**					
Yes	71	169	0.284 (0.210, 0.359)***	0.096 (− 0.058 0.251)				
Personal skills parents					0.083 (1, 215)	.774	.028***	.068***
No (RC)	56	113	0.255 (0.167, 0.343)***					
Yes	36	104	0.274 (0.173, 0.376)***	0.020 (− 0.114, 0.154)				
Parents’ self-confidence					4.389 (1, 215)	.037*	.027***	.067***
No (RC)	73	180	0.229 (0.156, 0.302)***					
Yes	21	37	0.397 (0.255, 0.539)***	0.168 (0.010, 0.325)*				
Attitudes/expectations toward parenting					1.486 (1, 215)	.224	.028***	.066***
No (RC)	81	202	0.250 (0.181, 0.319)***					
Yes	10	15	0.391 (0.173, 0.608)***	0.141 (− 0.087, 0.369)***				
Knowledge of typical child development					0.710 (1, 215)	.400	.028***	.067***
No (RC)	33	76	0.300 (0.190, 0.411)***					
Yes	59	141	0.243 (0.161, 0.324)***	− 0.058 (− 0.193, 0.078)				
Social network of family					0.068 (1, 215)	.795	.028***	.069***
No (RC)	66	153	0.269 (0.190, 0.348)***					
Yes	26	64	0.249 (0.124, 0.375)***	− 0.020 (− 0.167, 0.128)				
Relationship between parents					0.443 (1, 215)	.506	.029***	.066***
No (RC)	80	185	0.270 (0.201, 0.339)***					
Yes	14	32	0.217 (0.069, 0.366)**	− 0.053 (− 0.208, 0.103)***				
Relationship between parent and child				0.379 (1, 215)	.539	.028***	.069***	
No (RC)	45	107	0.243 (0.148, 0.337)***					
Yes	47	110	0.284 (0.190, 0.379)***	0.042 (− 0.092, 0.175)				
Mental health problems parents					0.148 (1, 215)	.701	.028***	.069***
No (RC)	71	159	0.256 (0.180, 0.333)***					
Yes	22	58	0.286 (0.154, 0.417)***	0.029 (− 0.121, 0.179)				
Empowerment					0.555 (1, 215)	.457	.028***	.068***
No (RC)	70	149	0.278 (0.201, 0.355)***					
Yes	22	68	0.221 (0.092, 0.350)***	− 0.057 (− 0.207, 0.093)				
Social/emotional support					0.450 (1, 215)	.503	.028***	.067***
No (RC)	46	101	0.286 (0.191, 0.382)***					
Yes	46	116	0.242 (0.152, 0.332)***	− 0.044 (− 0.174, 0.086)				
Well-being child					2.142 (1, 215)	.145	.028***	.065***
No (RC)	45	94	0.314 (0.218, 0.409)***					
Yes	48	123	0.220 (0.133, 0.306)***	− 0.094 (− 0.221, 0.033)				
Child skills					0.018 (1, 215)	.893	.028***	.069***
No (RC)	77	182	0.262 (0.189, 0.334)***					
Yes	15	35	0.273 (0.113, 0.434)***	0.012 (− 0.162, 0.186)				
Practical support					1.046 (1, 215)	.308	.028***	.067***
No (RC)	67	157	0.283 (0.206, 0.359)***					
Yes	26	60	0.207 (0.082, 0.333)**	− 0.075 (− 0.220, 0.070)***				
Motivation					1.517 (1, 215)	.219	.028***	.067***
No (RC)	88	209	0.271 (0.204, 0.339)***					
Yes	3	8	0.055 (− 0.283, 0.394)	− 0.216 (− 0.561, 0.130)				
**D: Delivery techniques**								
Modeling					0.842 (1, 184)	.360	.024***	.078***
No (RC)	52	112	0.253 (0.161, 0.346)***					
Yes	27	74	0.327 (0.199, 0.455)***	0.073 (− 0.084, 0.231)				
Role-playing					1.025 (1, 184)	.313	.025***	.075***
No (RC)	70	164	0.264 (0.186, 0.343)***					
Yes	9	22	0.384 (0.164, 0.603)***	0.120 (− 0.113, 0.352)				
Monitoring					1.898 (1, 184)	.170	.025***	.076***
No (RC)	61	145	0.307 (0.222, 0.392)***					
Yes	18	41	0.184 (0.031, 0.338)*	− 0.122 (− 0.297, 0.053)				
(Psycho) education					1.712 (1, 184)	.192	.025***	.072***
No (RC)	22	50	0.355 (0.216, 0.493)***					
Yes	58	136	0.249 (0.164, 0.333)***	− 0.106 (− 0.266, 0.054)				
Homework assignments					0.709 (1, 184)	.401	.024***	.076***
No (RC)	72	160	0.268 (0.189, 0.346)***					
Yes	7	26	0.374 (0.137, 0.612)**	0.107 (− 0.143, 0.357)				
Cognitive skills training					1.784 (1, 184)	.183	.024***	.078***
No (RC)	76	177	0.268 (0.192, 0.344)***					
Yes	4	9	0.504 (0.163, 0.846)**	0.236 (− 0.113, 0.585)				
Family group conferencing					0.246 (1, 184)	.620	.024***	.077***
No (RC)	77	184	0.276 (0.201, 0.351)***					
Yes	2	2	0.424 (− 0.161, 1.010)	0.148 (− 0.442, 0.739)				

# *Studies* number of studies, # *ES* number of effect sizes, *mean d* mean effect size (Cohen’s *d*), *CI* confidence interval, *β*_1_ estimated regression coefficient, *df* degrees of freedom, *level 2 variance* variance within studies, *Level 3 variance* variance between studies

*p < .05; **p < .01; ***p < .001

^+^ *p* < .1

^a^Omnibus test of al regression coefficients of the model

^b^ *p* value of the omnibus test

**Table 4:** Results of the moderator analyses for curative interventions (bivariate models)

Moderator variables	# Studies	# ES	Intercept/mean *d* (95% CI)	*β*_1_ (95% CI)	*F* (*df*1, *df*2)^a^	*p* ^b^	Level 2 variance	Level 3 variance
Overall effect	32	135	0.364 (0.227, 0.502)***					
**A: Study design characteristics**								
Publication year	32	135	0.396 (0.252, 0.540)***	− 0.013 (− 0.031, 0.006)	1.889 (1, 133)	.172	.144***	.060*
*Sample characteristics*								
Sample size	32	135	0.408 (0.267, 0.550)***	− 0.001 (− 0.002, 0.000)^+^	3.489 (1, 133)	.064^+^	.145***	.054*
Age of the parents	12	40	0.468 (0.218, 0.718)***	0.009 (− 0.046, 0.065)	0.111 (1, 38)	.741	.059***	.121***
Percentage cultural minorities	20	111	0.396 (0.270, 0.523)***	− 0.592 (− 1.239, 0.055)^+^	3.284 (1, 109)	.073^+^	.182***	.010
*Design characteristics*								
Research design					0.265 (1, 133)	.608	.148***	.063*
RCT (RC)	19	115	0.340 (0.171, 0.509)***					
Quasi-experimental	13	20	0.417 (0.173, 0.662)***	0.077 (− 0.220, 0.374)				
Pilot/feasibility study					1.523 (1, 133)	.219	.147***	.058^+^
No (RC)	29	119	0.333 (0.188, 0.478)***					
Yes	3	16	0.594 (0.201, 0.986)**	0.261 (− 0.157, 0.679)				
*Outcome characteristics*								
Follow-up period	22	85	0.339 (0.168, 0.509)***	0.005 (− 0.008, 0.017)	0.510 (1, 83)	.477	.095***	.074^+^
Type of outcome measure					3.241 (2, 132)	.042*	.134***	.059*
Self-report parents (RC)	14	72	0.216 (0.033, 0.398)***					
Official reports	26	39	0.498 (0.328, 0.668)***	0.282 (0.057, 0.508)*				
Other	4	24	0.200 (− 0.075, 0.474)	− 0.016 (− 0.265, 0.233)				
**B: Contextual factors**								
Delivery setting					0.202 (2, 124)	.817	.151***	.060*
Combination of locations (RC)	5	63	0.350 (0.028, 0.672)***					
Home/ambulant	13	36	0.431 (0.217, 0.644)***	0.081 (− 0.305, 0.467)				
Treatment center	9	28	0.479 (0.220, 0.737)***	0.129 (− 0.273, 0.531)				
Post-/prenatal								
Postnatal (RC)	29	132	0.359 (0.215, 0.502)***		0.117 (1, 133)	.733	.149***	.063*
Prenatal and postnatal	3	3	0.459 (− 0.102, 1.020)	0.100 (− 0.479, 0.679)				
Type of intervention					0.119 (5, 129)	.988	.150***	.084*
Family-based/multisystemic (RC)	14	60	0.346 (0.132, 0.561)**					
Home visitation intervention	6	14	0.344 (− 0.009, 0.697)^+^	− 0.003 (− 0.416, 0.411)				
Parent training	6	11	0.415 (0.025, 0.806)*	0.069 (− 0.376, 0.515)				
Substance abuse intervention	3	18	0.385 (− 0.025, 0.795)^+^	0.039 (− 0.424, 0.501)				
Crisis intervention	2	2	0.335 (− 0.376, 1.047)	− 0.011 (− 0.754, 0.732)				
Cognitive behavioral therapy	2	30	0.445 (0.123, 0.767)**	0.098 (− 0.178, 0.375)				
Specific individual interventions					0.375 (6, 128)	.894	.148***	.081*
Other interventions (RC)	17	100	0.402 (0.200, 0.603)***					
Parent–child interaction therapy	3	9	0.270 (− 0.184, 0.724)	− 0.132 (− 0.629, 0.365)				
FGC/FGDM	5	8	0.133 (− 0.269, 0.535)	− 0.268 (− 0.718, 0.181)				
Intensive fam preservation serv	2	2	0.335 (− 0.369, 1.040)	− 0.066 (− 0.799, 0.667)				
Incredible years	2	4	0.461 (− 0.167, 1.089)	0.060 (− 0.600, 0.719)				
MST (CAN/BSF)	2	10	0.545 (0.023, 1.068)*	0.143 (− 0.417, 0.703)				
Project 12-ways	2	2	0.470 (− 0.235, 1.176)	0.069 (− 0.665, 0.802)				
Age of the child					0.163 (3, 122)	.921	.160***	.074*
Primary school (6–12) (RC)	17	98	0.375 (0.176, 0.573)***					
Unborn child/baby (≤ 2)	3	3	0.460 (− 0.127, 1.047)	0.086 (− 0.534, 0.706)				
Infant/toddler (2–5)	7	17	0.267 (− 0.045, 0.579)^+^	− 0.108 (− 0.478, 0.262)				
High school (≥ 12)	1	8	0.391 (− 0.236, 1.018)	0.016 (− 0.641, 0.674)				
**C: Structural elements**								
Type of worker					0.543 (1, 130)	.463	.153***	.065
Professionals (RC)	26	125	0.353 (0.201, 0.506)***					
Not only professionals	4	7	0.524 (0.093, 0.955)*	0.170 (− 0.287, 0.627)				
Duration					0.709 (3, 122)	.548	.157***	.063*
0–6 months (RC)	15	91	0.453 (0.251, 0.656)***					
7–12 months	5	15	0.311 (− 0.047, 0.668)^+^	− 0.143 (− 0.546, 0.261)				
13–24 months	5	19	0.190 (− 0.120, 0.500)	− 0.263 (− 0.633, 0.107)				
24 months	1	1	0.437 (− 0.571, 1.446)	− 0.016 (− 1.044, 1.013)				
Minimum duration	9	30	0.433 (0.185, 0.680)**	− 0.005 (− 0.022, 0.012)	0.371 (1, 28)	.547	.023	.084^+^
Maximum duration	23	121	0.407 (0.266, 0.548)***	− 0.002 (− 0.005, 0.001)	1.601 (1, 119)	.208	.163***	.031
Average number of sessions	13	39	0.356 (0.198, 0.513)***	− 0.009 (− 0.027, 0.008)	1.146 (1, 37)	.291	.070***	.026
Interval sessions					0.387 (4, 111)	.818	.172***	.062^+^
Weekly (RC)	7	80	0.430 (0.166, 0.695)***					
Multiple sessions a week	5	14	0.452 (0.086, 0.817)*	0.021 (− 0.430, 0.472)				
Every other week/monthly	1	2	0.885 (− 0.064, 1.835)^+^	0.455 (− 0.530, 1.440)				
Ascending/descending intensity	5	19	0.344 (0.027, 0.662)*	− 0.086 (− 0.499, 0.327)				
Every 3 months	1	1	0.108 (− 0.943, 1.159)	− 0.323 (− 1.407, 0.761)				
**C: Specific intervention practices (content)**								
Parenting skills					5.089 (1, 133)	.026*	.150***	.040
No (RC)	10	45	0.190 (− 0.006, 0.387)^+^					
Yes	23	90	0.430 (0.290, 0.569)***	0.239 (0.029, 0.449)*				
Personal skills parents					3.063 (1, 133)	.082^+^	.142***	.057*
No (RC)	10	20	0.177 (− 0.073, 0.559)^+^					
Yes	22	115	0.440 (0.280, 0.600)***	0.263 (− 0.034, 0.559)^+^				
Parents’ self-confidence					1.252 (1, 133)	.265	.144***	.062*
No (RC)	26	120	0.327 (0.174, 0.480)***					
Yes	6	15	0.525 (0.209, 0.842)**	0.199 (− 0.153, 0.550)				
Attitudes/expectations parenting					1.643 (1, 133)	.202	.146***	.061*
No (RC)	31	105	0.354 (0.215, 0.492)***					
Yes	2	30	0.529 (0.240, 0.818)***	0.176 (− 0.095, 0.446)				
Knowledge of typical child development					1.059 (1, 133)	.305	.152***	.054
No (RC)	27	124	0.393 (0.247, 0.538)***					
Yes	5	11	0.194 (− 0.159, 0.547)	− 0.199 (− 0.581, 0.183)				
Social network of family					0.063 (1, 133)	.803	.147***	.065*
No (RC)	17	95	0.348 (0.154, 0.541)***					
Yes	15	40	0.383 (0.182, 0.585)***	0.035 (− 0.244, 0.315)				
Relationship between parents					0.491 (1, 133)	.485	.144***	.070*
No (RC)	28	92	0.379 (0.232, 0.526)***					
Yes	6	43	0.297 (0.058, 0.536)*	− 0.082 (− 0.315, 0.150)				
Relationship between parent and child				1.524 (1, 133)	.219	.151***	.055	
No (RC)	18	71	0.424 (0.259, 0.589)***					
Yes	15	64	0.296 (0.124, 0.469)***	− 0.127 (− 0.332, 0.077)				
Mental health problems parents					3.613 (1, 133)	.059^+^	.142***	.056*
No (RC)	20	95	0.258 (0.086, 0.431)**					
Yes	12	40	0.521 (0.309, 0.733)***	0.263 (− 0.011, 0.536)^+^				
Empowerment					2.854 (1, 133)	.093^+^	.151***	.047^+^
No (RC)	20	111	0.440 (0.282, 0.599)***					
Yes	12	24	0.203 (− 0.025, 0.431)^+^	− 0.237 (− 0.515, 0.041)^+^				
Social/emotional support					4.581 (1, 133)	.034*	.149***	.042
No (RC)	24	121	0.296 (0.155, 0.437)***					
Yes	8	14	0.649 (0.355, 0.942)***	0.352 (0.027, 0.678)*				
Well-being child					3.600 (1, 133)	.060^+^	.144***	.053^+^
No (RC)	14	23	0.272 (0.108, 0.435)**					
Yes	18	112	0.539 (0.313, 0.766)***	0.268 (− 0.011, 0.547)^+^				
Skills child					2.282 (1, 133)	.133	.146***	.058*
No (RC)	26	94	0.327 (0.183, 0.471)***					
Yes	7	41	0.499 (0.276, 0.723)***	0.172 (− 0.053, 0.398)				
Practical support					0.311 (1, 133)	.578	.146***	.066*
No (RC)	20	108	0.335 (0.160, 0.510)***					
Yes	12	27	0.417 (0.185, 0.649)***	0.082 (− 0.209, 0.373)				
Motivation					2.2021 (1, 133)	.157	.148***	.056^+^
No (RC)	26	92	0.400 (0.256, 0.544)***					
Yes	7	43	0.239 (0.019, 0.459)*	− 0.161 (− 0.385, 0.063)				
**D: Delivery techniques**								
Modeling					0.199 (1, 127)	.656	.160***	.069*
No (RC)	22	116	0.378 (0.209, 0.548)***					
Yes	7	13	0.294 (− 0.038, 0.627)^+^	− 0.084 (− 0.457, 0.289)				
Role-playing					0.091 (1, 127)	.764	.158***	.072*
No (RC)	20	109	0.347 (0.169, 0.525)***					
Yes	9	20	0.399 (0.108, 0.691)**	0.052 (− 0.290, 0.394)				
Monitoring					1.073 (1, 127)	.302	.161***	.060^+^
No (RC)	23	116	0.323 (0.160, 0.485)***					
Yes	6	13	0.517 (0.183, 0.851)**	0.194 (− 0.177, 0.565)				
(Psycho) education					1.144 (1, 127)	.287	.162***	.061
No (RC)	22	85	0.391 (0.234, 0.549)***					
Yes	8	44	0.266 (0.039, 0.494)*	− 0.125 (− 0.357, 0.106)				
Homework assignments					1.951 (1, 127)	.165	.161***	.060^+^
No (RC)	23	79	0.318 (0.160, 0.475)***					
Yes	7	50	0.479 (0.256, 0.702)***	0.162 (− 0.067, 0.390)				
Cognitive skills training					1.197 (1, 127)	.276	.165***	.056
No (RC)	23	77	0.326 (0.169, 0.483)***					
Yes	8	52	0.449 (0.232, 0.667)***	0.124 (− 0.100, 0.348)				
Family group conferencing					1.529 (1, 127)	.218	.154***	.070*
No (RC)	22	112	0.412 (0.241, 0.583)***					
Yes	7	17	0.189 (− 0.124, 0.502)	− 0.223 (− 0.580, 0.134)				

# *Studies* number of studies, # *ES* number of effect sizes, mean *d* mean effect size (Cohen’s *d*), *CI* confidence interval, *β*_1_ estimated regression coefficient, *df* degrees of freedom, *level 2 variance* variance within studies, *level 3 variance* variance between studies

*p < .05; **p < .01; ***p < .001

^+^ *p* < .1

^a^Omnibus test of al regression coefficients of the model

^b^ *p* value of the omnibus test

*Study design characteristics* For preventive interventions, a trend significant moderating effect was found for research design (larger effect sizes were found in quasi-experimental designs (*d* = .374) versus RCTs (*d* = .226)), and a significant positive moderating effect was found for follow-up period (intervention effects increased as follow-up length increased). For curative interventions, a trend significant negative moderating effect was found for sample size (larger sample sizes yielded smaller effect sizes), and a trend significant negative moderating effect was found for percentage of cultural minorities in samples (higher percentages of minorities yielded smaller effect sizes). A significant effect was found for type of outcome measure (larger effect sizes were found for official reports compared to self-reports of parents).

*Contextual factors* For preventive interventions, a significant moderating effect was found for the type of intervention: The effect of parent training interventions (*d* = .428) and substance abuse interventions (*d* = 1.852) were significantly higher than home visitation interventions (*d* = .210). The types of preventive interventions that were effective in preventing child maltreatment were: home visitation interventions (*d* = .210), parent training interventions (*d* = .428), family-based/multisystemic interventions (*d* = .343), substance abuse interventions (*d* = 1.852) and combined interventions (*d* = .174). Before-school interventions (*d* = .148), general prevention interventions (*d* = .024), and crisis interventions (*d* = .407) did not have a significant effect on preventing child maltreatment (the latter probably due to lack of power). Types of curative interventions that were effective in reducing child maltreatment were home visitation interventions (*d* = .344; trend significant), parent training interventions (*d* = .415), family-based/multisystemic interventions (*d* = 0.346), substance abuse interventions (*d* = .385; trend significant), and cognitive behavioral therapy (*d* = .445). Crisis interventions (*d* = .335) did not have a significant effect on reducing child maltreatment, probably due to lack of power.

Further, the moderator analysis showed that the effect of any of the specific individual interventions did not significantly deviate from the effect of the reference category (i.e., other interventions). Specific individual interventions were tested as a separate category only if the effect of the intervention has been examined in at least two studies. Specific individual interventions with a (trend) significant effect on preventing or reducing child maltreatment were: MST-CAN/BSF (*d* = .545), Triple P (*d* = .417), ACT-Parent’s Raising Safe Kids Program (*d* = .377), and Healthy Start (*d* = .339).

*Structural elements* For preventive interventions, higher effect sizes were found for interventions delivered by professionals only (*d* = .334) compared to interventions delivered by professionals and others (such as paraprofessionals or non-professionals; *d* = .184). Higher effect sizes were found for interventions with a shorter duration (*d* = .361 for 0–6 months) compared to interventions with a longer duration (*d* = −.051 for 7–12 months; *d* = .236 for 13–24 months (trend significant difference); and *d* = .190 for longer than 24 months). For curative interventions, none of the structural elements were significantly related to effect size.

*Specific interventions practices and delivery techniques* For preventive interventions, larger effect sizes were found for interventions focusing on increasing the self-confidence of parents (*d* = .397 versus *d* = .229). For curative interventions, larger effect sizes were found for improving parenting skills (*d* = .430 versus *d* = .190), improving personal skills of parents (*d* = .440 versus *d* = .177; trend significant difference), addressing mental health problems parents (*d* = .521 versus *d* = .258; trend significant difference), providing social and/or emotional support (*d* = .649 versus *d* = .296), and improving child well-being (*d* = .539 versus *d* = .272; trend significant difference). Smaller effect sizes were found for interventions focusing on empowerment (*d* = .203 versus *d* = .440; trend significant difference).

### Moderator Analyses Multiple Moderator Models

Finally, the moderators that were significant in the bivariate models were tested jointly in a multiple moderator model for preventive and curative interventions separately. In this way, the unique contribution of moderators to the prediction of effect size could be examined (see Tables 5 and 6). In the multiple moderator model for preventive interventions, heterogeneity in intervention effects was particularly explained by follow-up duration, parent training interventions (versus other interventions; trend significant), substance abuse interventions (versus other interventions), and a focus on increasing self-confidence of parents (versus interventions without this focus). In the multiple moderator model for curative interventions, heterogeneity in intervention effects was particularly explained by outcome measure, a focus on improving parenting skills (versus interventions without this focus), and providing social/emotional support (versus interventions without this practice; trend significant). In both models, multicollinearity did not seem to be a problem, since all variance inflation factors were below 1.302 and all tolerance statistics were above 0.768.

**Table 5:** Results of the multiple moderator model for preventive interventions

Moderator variables	*β* (SE)	95% CI	*t*-statistic
Intercept	0.111 (0.094)	− 0.077, 0.299	1.182
Control variables			
Follow-up duration	0.002 (0.001)*	0.000, 0.003	2.595
Parent training (vs. other intervention types)	0.180 (0.100)^+^	− 0.019, 0.379	1.805
Substance abuse intervention (vs. other intervention types)	1.404 (0.464)**	0.478, 2.329	3.025
Professional workers (vs. not only professionals)	0.119 (0.090)	− 0.062, 0.299	1.313
Intervention duration > 6 months (vs. 0–6 months)	− 0.119 (0.086)	− 0.290, 0.052	− 1.392
Parents’ self-confidence (vs. interventions without self-confidence in intervention content)	0.275 (0.123)*	0.029, 0.521	2.231
*F* (*df*1, *df*2)	6.117 (6, 69)***		
Level 2 variance	0.014***		
Level 3 variance	0.037***		

*Β* estimated regression coefficient, *SE* standard error, *CI* confidence interval, *F F*-statistic (omnibus test), *Level 2 variance* variance within studies, *Level 3 variance* variance between studies

*p < .05; **p < .01; ***p < .001

^+^ *p* < .100

**Table 6:** Results of the multiple moderator model for curative interventions

Moderator variables	*β* (SE)	95% CI	*t*-statistic
Intercept	− 0.029 (0.111)	− 0.248, 0.190	− 0.264
Control variables			
Official records (vs. other outcome types)	0.273 (0.107)*	0.061, 0.485	2.547
Parenting skills (vs. interventions without parenting skills in intervention content)	0.288 (0.100)**	0.089, 0.486	2.867
Social/emotional support (vs. interventions without support in intervention content)	0.264 (0.155)^+^	− 0.043, 0.571	1.700
*F* (*df*1, *df*2)	6.180 (3, 131)***		
Level 2 Variance	0.130***		
Level 3 Variance	0.028		

*Β* estimated regression coefficient, *SE* standard error, *CI* confidence interval, *F F*-statistic (omnibus test), *Level 2 variance* variance within studies, *Level 3 variance* variance between studies

*p < .05; **p < .01; ***p < .001

^+^ *p* < .100

## Discussion

The aim of the present meta-analysis was to gain insight into components contributing to the effectiveness of child maltreatment interventions. Overall, a small but significant effect was found of interventions aimed at preventing or reducing child maltreatment (*d* = .287), which is in line with findings of previously conducted meta-analyses on the effect of these interventions (e.g., Geeraerts et al. 2004; Filene et al. 2013; MacLeod and Nelson 2000). The results of the trim-and-fill analysis suggested that bias was present in the data, and therefore, a “corrected” overall effect was estimated, resulting in an effect size of *d* = 0.301. Because there are several methodological shortcomings regarding the trim-and-fill method (see the Limitations section), this corrected effect size should not be interpreted as a true effect size, but only as an indicator of (possible) bias in the data.

Larger effect sizes were found for curative interventions targeting maltreating parents (*d* = .364) than for preventive interventions targeting at-risk families/the general population (*d* = .263), but this difference did not reach significance. Euser et al. (2015) did find a significantly higher effect for interventions aimed at reducing child maltreatment in maltreating families than for interventions aimed at preventing child maltreatment in at-risk families/the general population. The finding that curative interventions are more effective than preventive interventions may be explained by a lower prevalence of child maltreatment in at-risk families/the general population than in maltreating families, making it “more difficult” to find significant differences between intervention and control groups (because of lower statistical power) and consequently, to prove the effectiveness of an intervention. Furthermore, the detection of child maltreatment in the general population/at-risk families is particularly difficult when official records are used, as many maltreatment occurrences are not officially reported to child protection services. So, in primary studies examining the effect of a preventive intervention and in which official records are used produce rather small effect sizes. Therefore, relatively large research groups and long follow-up periods are needed to increase the power. This may also be a possible explanation for the significant effect of the variable follow-up period (higher effect sizes were produced in longer follow-up periods): the longer the follow-up period, the higher the prevalence of child abuse in the research groups, the more likely a possible effect is detected.

### Study Design Characteristics

For preventive interventions, higher effect sizes were found for studies with quasi-experimental designs (*d* = .347) than for RCTs (*d* = .226; trend significant difference). Previous meta-analyses that included only RCT designs generally showed smaller effect sizes than meta-analysis including both RCTs and quasi-experimental designs, which is in line with the findings of our meta-analysis. For example, Pinquart and Teubert (2010) found a very small effect (*d* = .13) of RCTs on parenting interventions for families with newborns. Euser et al. (2015) also found a very small effect (*d* = 0.13) of RCTs on interventions for preventing or reducing child maltreatment. Ultimately, exclusively reviewing RCT studies is desirable, because effects of child maltreatment interventions can best be determined in RCT’s, as random assignment of participants to an experimental and a control group (theoretically) equalizes both groups on all other variables. Therefore, RCT’s are considered to be the most powerful study design in intervention research. However, at present, it is too early for such a review, because only a few RCTs have been performed on the effectiveness of child maltreatment interventions and their components. Consequently, a lot of essential information would be missing in a review of only RCTs and as a result, little knowledge could be obtained about effective components. Additionally, when matching procedures are properly applied in quasi-experimental studies, reasonable control groups can be formed so that generalization of results is quite feasible and realistic.

Further, for preventive interventions, larger positive intervention effects were found at later follow-up than at immediate post-intervention, which may be attributed to sleeper effects of interventions (Maurer et al. 2007). Sleeper effects in the context of child maltreatment interventions imply that positive intervention effects—at least to some extent—need time to emerge after interventions have ended. For example, parents may be unsure about applying newly learned parenting practices in their own home environment without being helped or supervised and, consequently, time passes until parents are able to apply these practices effectively. As soon as parents have practiced and acquired more confidence, the newly learned practices may be reinforced by positive responses of the child, other family members, and/or members of the social network of the family. It takes a considerable amount of time until positive parenting practices sink in with parents, simply because replacing adverse parenting practices and/or beliefs with positive parenting strategies cannot be expected to occur within a short time period. In general, child maltreatment interventions aim for a *sustained* change in parent–child interactions over time by improving parenting practices and/or beliefs, so that (the recurrence of) child maltreatment is prevented. Therefore, it is important in primary research to thoroughly conduct follow-up evaluations of considerable length, as the true effects of child maltreatment interventions may be particularly expressed in follow-up rather than in post-treatment evaluations.

For curative interventions, a moderating effect was found for type of outcome (smaller effect sizes were found for studies using self-report data obtained from parents compared to studies using official reports). This may be explained by two difficulties that arise when using self-report methods, such as interviews or questionnaires. First, it may be difficult for parents to be honest about the way they raise their children and to admit any neglectful or abusive parenting practices. As a result, parents may give socially desirable answers. Second, parents may be biased toward presenting a more favorable image of their own parenting behavior (see, for instance, Schwarz et al. 1985). Both issues may result in an underestimation of the true number of maltreatment occurrences, which in turn influences the magnitude of effect sizes. On the other hand, it can be assumed that official reports also lead to an underrepresentation of child abuse because researchers found that many occurrences of child maltreatment do not appear in official records (e.g., Fergusson et al. 2000; Finkelhor 2008; MacMillan et al. 2003).

### Effective Components

As for intervention duration, we found that preventive interventions with a short duration (up to 6 months) were more effective than preventive interventions of longer duration, whereas for curative interventions, no significant effect of intervention duration was found. This finding is in line with findings of a meta-analysis on effective ingredients of prevention programs for youth at risk for juvenile delinquency, which also showed that preventive interventions of shorter duration were more effective than preventive interventions of longer duration (De Vries et al. 2015). In contrast, Euser et al. (2015) concluded in their meta-analysis that interventions with an average length (6–12 months) were most effective, but this conclusion seems too strong. There was hardly any absolute difference in mean effect between their 0–6 month category (*d* = .22) and their 6–12 month category (*d* = .23). The “*less is more”* effect in attachment-based interventions found by Bakermans-Kranenburg et al. (2003) seems also applicable to interventions aimed at reducing or preventing child maltreatment. However, intervention duration was no longer a significant predictor of effect size in the multiple moderator model, which implies that intervention duration does not make a unique contribution to the prediction of effect size above follow-up duration (in months), and whether or not interventions were a parent training intervention, a substance abuse intervention, and focusing on improving parents’ self-confidence.

Moreover, it seems to be important to adjust the intensity of an intervention to the level of risk present in a family. In case of high risk, it may be important to intervene intensively, whereas in case of low risk, less intensive interventions are appropriate. This principle is known as the risk principle of the risk–need–responsivity (RNR) model (e.g., Andrews and Bonta 2010a, b; Andrews et al. 1990). This model is widely used for preventing recidivism in the (juvenile) criminal justice system, as various meta-analyses have shown that judicial interventions aimed at behavioral change are most effective when delivered according to this model (Andrews et al. 1990; Andrews and Dowden 1999). It can be assumed that the RNR principles also apply to interventions aimed at preventing or reducing child maltreatment, because child maltreatment, like delinquent behavior, is caused by the accumulation of risk factors in multiple systems (Belsky 1980, 1993; Brown et al. 1998). Furthermore, many risk factors of delinquent behavior resemble risk factors for child maltreatment, like poverty, stress in the family, and psychiatric problems of parents, including alcohol or drug problems (Stith et al. 2009).

For preventive interventions, lager effect sizes were found for interventions focusing on increasing self-confidence of parents. For curative interventions, larger effect sizes were found for improving parenting skills, improving personal skills of parents (trend significant), addressing mental health problems of parents (trend significant), providing social and/or emotional support, and improving a child’s well-being (trend significant). These findings offer possibilities to improve interventions. For curative interventions, smaller effect sizes were found for interventions focusing on empowerment (trend significant). This finding is in line with studies reporting that children from high-risk families benefit less from the presence of protective factors (e.g., Luthar and Goldstein 2004; Miller et al. 1999; Vanderbilt-Adriance and Shaw 2008), indicating that interventions aimed at increasing or strengthening protective factors in high-risk families do not necessarily lead to a decrease in child maltreatement. Vanderbilt-Adriance and Shaw (2008) concluded in their review of studies on resilience that in high-risk families, there should be a focus on both strengthening protective factors and reducing risks, because there are limits to the amount of risk that can be mitigated.

### Implications for Policy and Clinical Practice

First, when implementing best practices, clinical professionals and policy makers should opt for interventions that produce the largest effects on preventing or reducing child maltreatment. Cognitive behavioral therapy, home visitation, parent training, family-based/multisystemic, substance abuse, and combined interventions were effective in preventing and/or reducing child maltreatment. Specific individual interventions with a (trend) significant effect on preventing or reducing child maltreatment that were examined in at least two independent studies were: MST-CAN/BSF (intensive family therapy), Triple P (a parent training), ACT-Parent’s Raising Safe Kids Program (a short-term parent training), and Healthy Start (a home visitation intervention). Implementing these (types of) interventions in clinical practice may therefore be fruitful.

Second, the effectiveness of existing interventions could be improved by integrating specific components associated with greater effectiveness into interventions, such as focusing on improving parenting skills and providing social and/or emotional support in curative interventions, and increasing self-confidence of parents in preventive interventions. For curative interventions, it seems important to screen for mental health problems in parents and if present, to address these problems in interventions. Interventions targeting substance-abusing parents appeared to be very effective, as well as interventions addressing other mental health problems. Furthermore, improving child well-being and providing social and/or emotional support seems to be effective components of interventions aimed at maltreating families.

### Limitations

Several limitations need to be discussed. First, a large amount of literature is available on the effectiveness of interventions aimed at preventing or reducing child maltreatment, and therefore, it is possible that we may have missed primary studies. However, we included a rather large number of studies in our meta-analysis (121 independent studies) and this led us to assume that our sample of studies is representative of all studies on the effect of child maltreatment interventions that have been conducted to date. Second, the reported information of the studies included in the meta-analysis was sometimes limited. A relatively large number of studies failed to report important information on intervention characteristics, such as specific intervention practices and delivery techniques.

A third limitation is related to the outcome measure that was assessed in primary studies. In some studies, it is assumed that a report, investigation, or a recurrence in child protection is indicative of abuse or neglect. However, the relationship between contact with the child protection system and maltreatment is not straightforward (Jenkins et al. 2017). Several studies showed that a large proportion of child maltreatment is not reported to child protection authorities (Cyr et al. 2013; Finkelhor et al. 2005, 2009). Population-based surveys showed that rates of maltreatment are more than ten times the rates of substantiated maltreatment in those same jurisdictions (e.g., Fergusson et al. 2000; Finkelhor 2008; MacMillan et al. 2003). Further, in many cases reported to child protection authorities, maltreatment is not substantiated.

Fourth, there are several methodological difficulties regarding the trim-and-fill method. The performance of the trim-and-fill method is limited when effect sizes prove to be heterogeneous (Peters et al. 2007; Terrin et al. 2003). In addition, the application of the trim-and-fill method could mean adding and adjusting for nonexistent effect sizes in response to funnel plots that are asymmetrical, simply because of random variation (Egger et al. 2001). Finally, Nakagawa and Santos (2012) mentioned that this method has originally been designed for meta-analytic reviews in which independence of effect sizes can be assumed. Despite these shortcomings, there is no best method for detecting and handling missing data in meta-analysis, and therefore, the results from the trim and fill method should be interpreted with caution. In the present study, we only used the trim-and-fill method to calculate a “corrected” overall effect. A fifth limitation is that several moderator analyses were based on a rather small number of effect sizes, implying a low statistical power in the moderator analyses. Finally, it should be kept in mind that the present review was mainly based on primary studies that were conducted in Western countries, and in particular the USA. Since countries differ in social and political climate, organization of child welfare health services, and in ethnic and cultural background of clients served by child welfare, it is questionable whether the present results are representative for nonwestern countries.

Despite these limitations, our study provides important knowledge for science and practice. Our findings show that interventions can be effective in preventing or reducing child maltreatment. The results of this meta-analysis can be used to improve existing interventions, for example by integrating effective components in interventions, or by developing new promising interventions comprising of the most effective components.

## Appendix 1

See Table 7.

**Table 7:** Characteristics of included studies

References(s)^a^	*N* ^b^	#ES	Name intervention^c^	AC child	Design^d^	Type of families served^e^
Akai et al. (2008)	48	3	My baby and me	≤ 2	QE	R
Andrews et al. (1982)	38	1	PCDC	≤ 2	RCT	R
Antle et al. (2009)	760	1	Solution-based casework	NS	QE	M
Arancena et al. (2009)	90	1	Home visitation program	≤ 2	RCT	R
Asscher et al. (2008)	105	3	Home-Start	2–5	QE	R
Barnet et al. (2002)	147	1	Home visitation program	≤ 2	RCT	R
Barnet et al. (2007)	63	1	CB home visitation program	≤ 2	RCT	R
Barth et al. (1988)	60	2	CPEP	≤ 2	RCT	R
Barth (1991)	191	2	CPEP	≤ 2	RCT	R
Berzin et al. (2008)	45–60	3	FGDM	2–5	RCT	R/M
Black et al. (1994)	43	1	Home visitation program	≤ 2	RCT	R
Bouwmeester-Landweer (2006)	448–469	5	Stevig ouderschap [*Solid Parenting*]	≤ 2	RCT	R
Brayden et al. (1993)	263	3	Prenatal + pediatric health services	≤ 2	RCT	R
Britner and Reppucci (1997)	535	1	Parent education program	≤ 2	QE	R
Brooten et al. (1986)	79	1	Home follow-up care	≤ 2	RCT	R
Bugental et al. (2002)	73	2	HS/HS + cognitive component	≤ 2	RCT	R
Bugental and Schwartz (2009)	80	2	HS + cognitive component	≤ 2	RCT	R
Casanueva et al. (2008)	588	1	Parenting services	6–12	QE	M
Casiro et al. (1993)^f^	100	1	Homemaker services	≤ 2	RCT	R
Chaffin et al. (2004)	110	4	PCIT/Enhanced PCIT	6–12	RCT	R
Chaffin et al. (2011)	76	1	PCIT	2–5	RCT	R
Crampton and Jackson (2007)	94	1	FGDM	NS	QE	M
Culp et al. (2004)	263	1	CBFRS home visitation	≤ 2	QE	GP
Dakof et al. (2010)^f^	62	5	Engaging moms program	NS	RCT	M
Dawe and Harnett (2007)	41–42	2	Brief parenting skills training/PUP	2–5	RCT	R
Dawson et al. (1989)	111	1	Parent support home visits + parent groups	≤ 2	QE	R
Dew and Breakey (2014)	4466	1	Hawaii’s HS program	≤ 2	QE	R
Donohue et al. (2014)	72	4	Family behavior therapy	2–5	RCT	M
Dubowitz et al. (2009)	558	4	SEEK	6–12	RCT	R
Dubowitz et al. (2012)	894–1119	3	SEEK	6–12	QE	GP
Duggan et al. (2004)	541	7	Hawaii HS program	NS	RCT	R
Duggan et al. (2007)	249–297	7	HF Alaska	≤ 2	RCT	R
DuMont et al. (2008) (2) DuMont et al. (2011)	104–1173	10	HF New York	≤ 2	RCT	R/M
Easterbrooks et al. (2012)	512	2	HF Massachusetts	≤ 2	RCT	R
Falconer et al. (2011)	1045–1147	2	HF Florida	≤ 2	QE	R
Fennell and Fishel (1998)	18		STEP	6–12	RCT	M
Fergusson et al. (2005) (2) Fergusson et al. (2013)	370–391	4	Early start program	≤ 2	RCT	R
Fraser et al. (2000) (2) Armstrong and Morris (2000)	124–160	2	Home visitation program	≤ 2	RCT	R
Fujiwara et al. (2011)	115	1	Group Triple P	6–12	QE	R
Galanter et al. (2012)	63	1	PCIT	6–12	QE	R
Gershater-Molko et al. (2002)	82	1	Project SafeCare	2–5	QE	M
Gessner (2008)	2013	1	HF Alaska	≤ 2	QE	R
Gray et al. (1977)	50	2	Pediatric intervention	≤ 2	RCT	R
Green t al. (2014a)	108	1	Early head start	2–5	RCT	R
Green et al. (2014b)	763	1	HF Oregon	≤ 2	RCT	R
Guterman et al. (2013)	101	4	Parent aide services	6–12	RCT	R
Guthrie et al. (2009)^f^	72	1	Touchpoints training + home visiting	≤ 2	QE	R
Hans et al. (2013)	221	1	Community doula support	≤ 2	RCT	R
Harden et al. (2012)	531	1	Early head start	≤ 2	RCT	R
Harder (2005)	134–158	4	Parent aide program	2–5	QE	M
Hardy and Streett (1989)	263	1	Children and youth program	≤ 2	RCT	R
Honig and Morin (2001)	49–65	2	TPBP	≤ 2	QE	R
Hurlburt et al. (2013)	75–303	4	Incredible years	2–5	RCT	R
Huxley and Warner (1993)	40	1	Community infant project	≤ 2	QE	R
Jacobs et al. (2016)	594	2	HF Massachusetts	≤ 2	RCT	R
Javier et al. (2016)^f^	24	1	Incredible years School Age	6–12	RCT	R
Johnston et al. (2006)	207	2	Healthy steps/healthy steps + prepare	≤ 2	QE	GP
Jones Harden (2012)	927–948	2	Early head start	≤ 2	RCT	R
Jouriles et al. (2010)	35	4	Project support	6–12	RCT	M
Kagitcibasi et al. (2001)	217–225	2	TEEP	2–5	RCT	R
Kan and Feinberg (2014)	169	1	Family foundations program	≤ 2	RCT	R
Kirk and Griffith (2004)	515–5006	2	Intensive FPS	NS	QE	R
Knox et al. (2011)	92	3	ACT-raising safe Kids	2–5	QE	R
Knox et al. (2013)	84	1	ACT-raising safe kids	2–5	RCT	R
Kolko (1996)	27–30	56	CBT/family therapy	6–12	RCT	M
Lam et al. (2009)^f^	20	18	PSBCT	6–12	RCT	M
Larson (1980)	63	1	Home visits	≤ 2	QE	R
Lealman et al. (1983)	302–312	2	Prevention program	≤ 2	QE	R
LeCroy and Krysik (2011)	168	2	HF Arizona	≤ 2	RCT	R
Leijten et al. (2012)	78	2	PCTT	≥ 12	RCT	R
Letarte et al. (2010)	35	2	Incredible years	6–12	QE	M
Linares et al. (2006)	99–108	2	Incredible years	6–12	RCT	M
Love et al. (2005)	459–932	3	Early head start	≤ 2	RCT	R
Lowell et al. (2011)	157	4	Child First	≤ 2	RCT	R
Lutzker and Rice (1984)	97	1	Project 12-ways	≤ 2	QE	M
MacMillan et al. (2005)	163	6	Home visitation program	6–12	RCT	M
Marcenko and Spence (1994) (2) Marcenko et al. (1996)	187–197	2	Prevention program	≤ 2	RCT	R
McKelvey et al. (2012)	227	1	Thrive program	≤ 2	QE	R
Mejdoubi et al. (2015)	332	1	NFP	≤ 2	RCT	R
Minkovitz et al. (2003) (2) Minkovitz et al. (2007)	1308–2144	8	HSYC	2–5	RCT/QE	GP
Norr et al. (2003)	154–477	3	REACH-Futures	≤ 2	RCT	R
Olds et al. (1986) (2) Olds et al. (1994) (3) Olds et al. (1997)	204–256	6	NFP	≤ 2	RCT	R
Oveisi et al. (2010)	224	1	SOS helps	6–12	RCT	GP
Paradis et al. (2013)	215	1	Building healthy children	≤ 2	RCT	R
Pataki and Johnson (2005)	1157	5	HF New York	≤ 2	RCT	R
Pennell and Burford (2000)	63	2	FGDM	NS	QE	M
Peterson et al. (2003)	99	4	7-level parenting model	2–5	QE	R
Portwood et al. (2011)	156–207	2	ACT-raising safe kids	NS	RCT	R
Posthumus et al. (2012)	132	6	Incredible years	2–5	QE	R
Puffer et al. (2015)	270	1	PMD	2–5	RCT	R
Reynolds and Robertson (2003)	1408	1	CPC program	6–12	QE	R
Rodrigo et al. (2006)	290	2	Apoyo personal y familiar	NS	QE	R
Rodriguez et al. (2010)	522	4	HF New York	≤ 2	RCT	R
Runyon et al. (2010)	60	2	Parent–child CBT	6–12	RCT	M
Sanders et al. (2004)	74	4	Enhanced Triple P	2–5	RCT	R
Sanders et al. (2012)	104	4	Triple P online	2–5	RCT	R
Sawasdipanich et al. (2010)	116	2	Cognitive adjustment program	6–12	RCT	R
Schaeffer et al. (2013)^f^	43	2	MST-BSF	6–12	QE	M
Schilling et al. (2017)^f^	120	1	PriCARE	2–5	RCT	R
Scholer et al. (2010)	64	1	Play nicely program	2–5	RCT	GP
Schuler et al. (2000) (2) Nair et al. (2003)	161–171	1	Home intervention	≤ 2	RCT	R
Shapiro et al. (2014)	38–45	9	SSTP	≤ 2	RCT	R
Siegel et al. (1980)	99–119	1	Hospital + home support	≤ 2	RCT	R
Silovsky et al. (2011)	105	3	SafeCare	2–5	RCT	R
Stevens-Simon et al. (2001)	127	1	CAMP home visitation program	≤ 2	RCT	R
Suess et al. (2016)	73	1	STEEP	≤ 2	QE	R
Sundell and Vinnerljung (2004)	239	2	FGC	6–12	QE	M
Swenson et al. (2010)	86	8	MST-CAN	≥ 12	RCT	M
Szykula and Fleischman (1985)	48	1	Social learning treatment	6–12	RCT	M
Thomas and Zimmer-Gembeck (2011) (2) Thomas and Zimmer‐Gembeck (Thomas and Zimmer-Gembeck 2012)	76–198	4	PCIT	2–5	RCT	M
Velasques et al. (1984)	55	1	Special families care project	≤ 2	QE	R
Wagner and Clayton (1999)	311–312	2	Teen PAT/Teen PAT + CM	≤ 2	RCT	R
Wagner et al. (2002)	78–139	2	PAT	≤ 2	RCT	GP/R
Walton (1997)	137	1	IFPS	6–12	RCT	M
Walton (2001)	208	1	IFPS	6–12	RCT	M
Walton et al. (1992)	110	3	Family reunification services	6–12	RCT	M
Weigensberg et al. (2009)	650	1	FGDM	2–5	QE	M
Wesch and Lutzker (1991)	464	1	Project 12-ways	≤ 2	QE	M
Wolfe et al. (1988)	30	1	Parenting training	2–5	QE	R
Wood et al. (1988)	108	1	Homebuilders	6–12	QE	M
Zhai et al. (2013)	2807	5	Head Start	≤ 2	QE	R

*Pub. year* Publication year, *N* total sample size, #*ES* amount of effect sizes, *AC child* age category of the child at the start of the program, *Design* research design, *PCDC* parent child development centers, *CB* community-based, *CPEP* child–parent enrichment project, *FGDM* family group decision making, *HS* healthy start, *PCIT* parent–child interaction therapy, *CBFRS* community-based family resource service, *PUP* parents under pressure, *SEEK* safe environment for every kid, *HF* healthy families, *STEP* systematic training for effective parenting, *TPBP* teen parents and babies program, *TEEP* Turkish early enrichment project, *IFPS* intensive family preservation services, *ACT* adults and children together, *CBT* cognitive behavioral therapy, *PSBCT* parent skills with behavioral couples therapy, *PCTT* parents and children talking together, *FIRST* family information, referral and support team, *NFP* nurse family partnership, *HSYC* healthy steps for young children, *REACH* resources, education and care in the home, *PMD* parents make the difference, *CPC* child–parent center, *MST*-*BSF* multisystemic therapy—building stronger families, *PriCARE* primary child–adult relationship enhancement, *SSTP* stepping stones Triple P, *CAMP* Colorado adolescent maternity program, *STEEP* steps toward effective and enjoyable parenting, *FGC* family group conferences, *MST*-*CAN* multisystemic therapy for child abuse and neglect, *PAT* parent as teachers, *CM* case management, *CPS* child protective services, *NS* not specified, *QE* quasi-experimental, *RCT* randomized controlled trial, *R* risk group, *GP* general population, *M* maltreating parents

^a^When secondary or tertiary literature was available (studies making use of the same sample), this is indicated by ‘(2)’ or ‘(3)’

^b^When more than one sample size was present within a study (varied by effect size), the range was displayed (minimum N–maximum N)

^c^When more than one program was examined within a study, this was indicated by a slash (/) in between the different program names

^d^When more than one research design was used within a study, this was indicated by a slash (/) in between the different research designs

^e^When more than one type of sample was present within a study, this was indicated by a slash (/) in between the different types of samples

^f^Feasibility/pilot study
